# Educational climate seems unrelated to leadership skills of clinical consultants responsible of postgraduate medical education in clinical departments

**DOI:** 10.1186/1472-6920-10-62

**Published:** 2010-09-21

**Authors:** Bente Malling, Lene S Mortensen, Albert JJ Scherpbier, Charlotte Ringsted

**Affiliations:** 1 Department of Human Resources, Aarhus University Hospital, Skejby, Aarhus, Denmark; 2 Department of Internal Medicine, Regional Hospital, Viborg, Denmark; 3 Institute for Education, Faculty Health, Medicine and Life Sciences, Maastricht University, Netherlands; 4 Centre for Clinical Education, Copenhagen University and Capital Region, Rigshospitalet, Denmark

## Abstract

**Background:**

The educational climate is crucial in postgraduate medical education. Although leaders are in the position to influence the educational climate, the relationship between leadership skills and educational climate is unknown. This study investigates the relationship between the educational climate in clinical departments and the leadership skills of clinical consultants responsible for education.

**Methods:**

The study was a trans-sectional correlation study. The educational climate was investigated by a survey among all doctors (specialists and trainees) in the departments. Leadership skills of the consultants responsible for education were measured by multi-source feedback scores from heads of departments, peer consultants, and trainees.

**Results:**

Doctors from 42 clinical departments representing 21 specialties participated. The response rate of the educational climate investigation was moderate 52% (420/811), Response rate was high in the multisource-feedback process 84.3% (420/498). The educational climate was scored quite high mean 3.9 (SD 0.3) on a five-point Likert scale. Likewise the leadership skills of the clinical consultants responsible for education were considered good, mean 5.4 (SD 0.6) on a seven-point Likert scale. There was no significant correlation between the scores concerning the educational climate and the scores on leadership skills, r = 0.17 (p = 0.29).

**Conclusions:**

This study found no relation between the educational climate and the leadership skills of the clinical consultants responsible for postgraduate medical education in clinical departments with the instruments used. Our results indicate that consultants responsible for education are in a weak position to influence the educational climate in the clinical department. Further studies are needed to explore, how heads of departments and other factors related to the clinical organisation could influence the educational climate.

## Background

Postgraduate medical education (PGME) is a work-based education where learning and teaching takes place in a clinical context. On the one hand the young doctor (trainee) is under education and on the other hand he is a member of the staff in the clinical department. The clinical departments face the challenge of creating an educational environment that is supportive and learning-oriented [1] and at the same time meeting the demands from society to deliver efficient clinical services and research [2,3]. A common feature in PGME is the need for supervision and feedback among trainees and for being engaged in the responsibility for patients [4,5]. Furthermore, trainees need to be appreciated and valued as team members [5]. The challenge is to find a proper balance between involving trainees in patient treatment, and at the same time ensure patient safety and meet demands for a high production [6]. As the educational climate is perceived to have major influence on how trainees learn and perform, the quality of the educational climate has received increasing attention in the literature on PGME [1,5,7].

However, the concept educational climate has been used indiscriminately with culture, environment or learning context [8]. Nevertheless, all of these concepts include the same elements: atmosphere [7-9], the connection and personal relation with colleagues [5,9,10], openness to questions and appropriateness of supervision and feedback [1,5,9], and eventually the working conditions and organisation of the work [5,7,9]. The educational climate may differ considerably across clinical departments. Hence, in order to be able to change the educational climate, it is relevant to find out who contributes to this climate and to the specific ways of behavior and "living" in the clinical departments.

The organisational culture, according to Schein [11], is closely connected to leadership. Although all employees take part in developing the culture in a department, the leaders are in a position to deliberately influence the culture [11]. In PGME leaders can influence the educational climate by prioritising and attending a variety of activities in the department [2]and by role-modelling. Various authors have described the "good physician leader" as a necessity to the survival of teaching hospitals [10,12-14]. And since the responsibilities and obligations in managing PGME in the clinical departments are many, and has become a growing business [2], the nomination of leaders of PGME in clinical departments has been introduced in many countries [15-18].

However we do not know much of these educational leaders in PGME. In a previous study on the clinical consultant responsible for education (CRE) in clinical departments, we found that stakeholders expected the CRE to develop and improve the educational climate [19], which is in accordance with the description of desired competencies for a leader of PGME provided by Wong et al. [14]. They also indicated that structure of the educational program in the department was a necessary but not sufficient prerequisite for the educational climate. The CREs were expected to take the lead regarding educational matters [19]and stakeholders perceived the CREs to have fairly good administrative and leadership skills [20]. Hence, a positive relation between leadership skills of the CRE and the quality of the educational climate might be expected. Likewise, a positive relation between the CRE's administrative skills and the organisation of the daily clinical work could be expected. However, this inference cannot be drawn from current literature on PGME.

The purpose of this study was to explore the relationship between the educational climate in clinical departments and leadership skills in clinical consultants responsible for PGME.

## Methods

The study was a trans-sectional correlation study on the relationship between the educational climate in clinical departments and leadership performance of CREs. The unit of analysis is the clinical department.

### Context of the study

**Figure 1:**
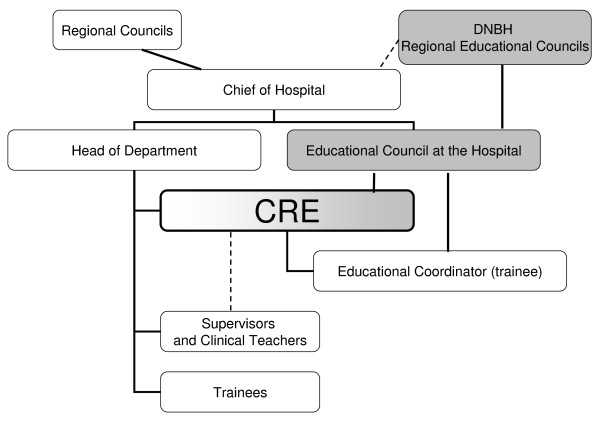
**Postgraduate medical education in Denmark - Organisational diagram**. The organisation of postgraduate medical education (PGME) in Denmark. The Danish National Board of Health (DNBH) sets rules and regulations regarding PGME. These are brought into effect by the Regional Educational Councils. Postgraduate medical education takes place in the clinical departments which are managed by the Regional Councils through the Chief of the Hospital. The Consultant responsible for education (CRE) is the pivotal link between the administrative and the educational line. The educational line is shown in gray boxes and the administrative line in white boxes.

Postgraduate medical education in Denmark is governed by the Danish National Board of Health. For clinical departments participating in PGME, it is mandatory to nominate one of the clinical consultants in the department to be leader of PGME in the department (CRE). The CRE has responsibility for a highly diverse group of trainees undergoing a number of different PGME programs at each clinical department. At the same time the position of a CRE is an important link between the administrative line and the educational line as shown in Figure 1. The CRE manages PGME in the clinical department and has both administrative and leader responsibilities ranging from organising the work in the department to assure all trainees get the proper education, through monitoring the evaluation of the trainees, to appointing and supervising the clinical teachers and supervisors in the department.

### Participants

This investigation took place in the Northern Educational Region in Denmark. This region covers one third of the country and includes both university and non-university hospitals. CREs from clinical departments with more than three consultants in addition to the head of the department and more than three trainees were eligible for inclusion.

CREs who had previously participated in a leadership course for CREs were excluded as they were included in another study also collecting multi-source feedback (MSF) data. Thus, a total of 79 CREs and their departments were eligible for inclusion. Participants were contacted by phone and informed about the study, the questionnaire on the educational climate and the MSF procedure. All gave informed consent. Confidentiality was guaranteed and participants were assured that it would be impossible to trace findings to individual participants, clinical departments or hospitals. The study was presented to the ethical committee for Viborg and Aalborg County. In our jurisdiction studies of this kind do not need approval.

### Questionnaire on the educational climate

Various instruments have been introduced to measure educational climate in PGME [9,21]. We chose a previously validated Danish instrument, derived from the Postgraduate Hospital Educational Environment Measure (PHEEM) instrument [9,21]. In this study we adjusted the instrument to comprise answers from all doctors in the department, thus including both specialists and trainees. The assumption was that all doctors in a department have an opinion about the educational climate [11]. The questionnaire comprised 36 statements divided into three main areas concerning the educational climate: 1) Learning opportunities, 2) Supervision and feedback, and 3) Organisation of work. A five-point Likert scale was used (1 = "totally disagree" and 5 = "totally agree" or best score). An e-mail based electronic system (Enalyzer^®^) was used to collect data.

### MSF on leadership skills

Multi-source feedback is a widely accepted tool for measuring leadership skills [22-24]. In this study we used an MSF instrument previously developed to specifically evaluate leadership skills of CREs [20]. Multi-source feedback scores were collected from heads of department, consultants and trainees [20]. The MSF instrument comprised 69 statements divided into four categories: 1)Technical skills, referring to the proficiency in specific leadership methods and processes; 2) Human skills, including the ability to work with and through people to meet goals; 3) Citizenship behaviour referring to professionalism regarding interpersonal, organisational and job/task performance; 4) Administrative skills, involving knowledge of the planning, organizing and coordinating of tasks [20]. Leaders' skills are normally perceived as two-dimensional with a leadership dimension and a management dimension. Accordingly, the statements in technical skills, human skills and citizenship behavior comprise leadership skills while administrative skills refer to management. Each statement was scored on a seven-point Likert scale (1 = "not at all" and 7 = "always" or best score). The option "not able to answer" was provided. The CRE chose at least three consultants and three trainees in the department to secure anonymity in addition to the head of department. Enalyzer^® ^was used to collect data

### Statistics

Mean scores of educational climate and MSF were calculated. If an item score was missing it was replaced by a mean of all other scores in the same category from the same respondent, provided that more than half of the items in the category were scored. If scores on more than half of the items were missing the respondent was excluded from further analysis. Separate mean scores were calculated for each of the three categories in the educational climate questionnaire, and separated into mean score from specialists and from trainees. Similarly, separate mean scores were calculated for the two overall categories "leadership skills" and "management skills" in the MSF procedure.

The overall educational climate score and scores for the three categories in the educational climate questionnaire were correlated to the overall MSF scores and the scores for leadership skills and management skills, respectively using Pearson's correlation coefficient. Scores from trainees and specialists were compared using Oneway ANOVA. A p-value < 0.05 was considered significant.

Descriptive statistics were used to examine whether characteristics of study sample were comparable to the background population. For this purpose the departments were categorised according to specialty type into 1) cognitive specialties (internal medicine and subspecialties, paediatrics, dermatology, oncology, psychiatry and neurology), 2) surgical specialties (surgery, orthopaedic surgery, urology, gynaecology, ophthalmology, otology, thoracic surgery, vascular surgery, brain surgery) and 3) technical specialties (anaesthesiology, radiology, all laboratory specialties).

## Results

**Figure 2:**
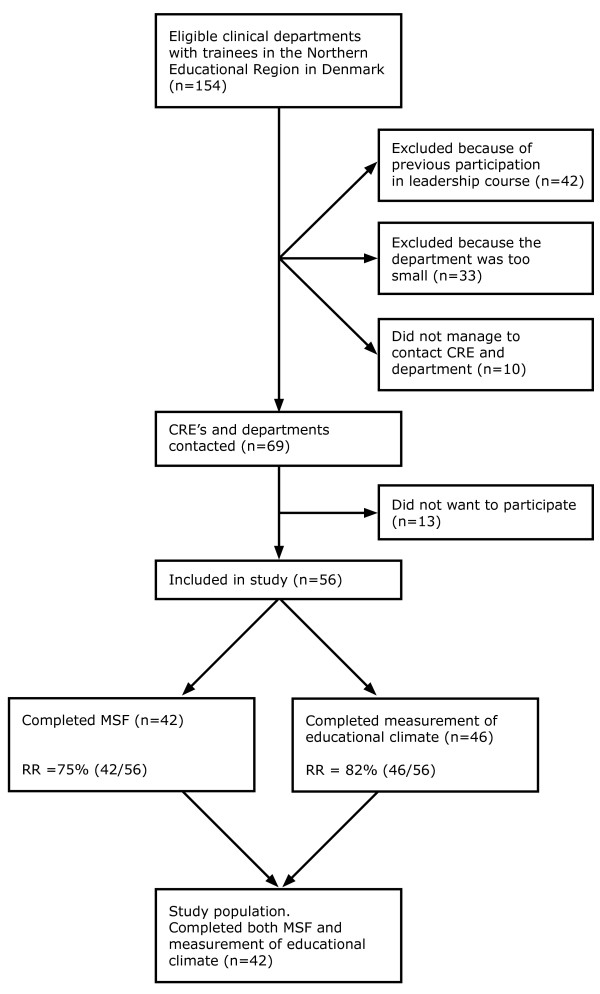
**Study population**. The number of consultants responsible for postgraduate medical education in clinical department (CRE) included in the multi-source feedback process (MSF) and the number of departments included in the measurement of the educational climate including response rates (RR).

Figure 2 show how the 154 eligible departments in the Northern Educational Region in Denmark resulted in a study population of 56 departments. Participants represented 21 of the 36 specialties in Denmark. The distribution between cognitive, surgical and technical departments in the study population did not reflect the background population mainly because many of the technical specialty departments (especially the laboratory departments) were too small to participate in the MSF procedure (Table 1). The proportion of cognitive and surgical departments was the same in the study group compared to the background population.

**Table 1:** Distribution of specialties in the background and the study population.

	*Specialties*			*Total*
	**Cognitive**	**Surgical**	**Technical**	

**Eligible clinical departments**	60 (39%)	48 (31%)	46 (30%)	154
**Excluded - previous leadership course**	13 (31%)	16 (38%)	13 (31%)	42
**Excluded - department too small**	9 (27%)	5 (15%)	19 (58%)	33
**Included in study**	27 (48%)	21 (38%)	8 (14%)	56

Distribution of clinical departments according to specialty in the total population, study sample and excluded departments in actual numbers and percentages of the total sum. Cognitive specialties include internal medicine and subspecialties, paediatrics, dermatology, oncology, psychiatry and neurology. Surgical specialties include surgery, orthopaedic surgery, urology, gynaecology, ophthalmology, otology, thoracic surgery, vascular surgery and brain surgery. Technical specialties include anaesthesiology, radiology and all laboratory specialties.

An average of ten doctors answered the questionnaire on the educational climate in each department. The response-rate was 52% (420/811). In the MSF-process each CRE had ten respondents on average (a total of 420 respondents answered the MSF). The response rate was high 84.3% (420/498). Mean educational climate and MSF scores are shown in Table 2. There was no significant correlation between total mean scores of the educational climate and the MSF scores, r = 0.17, p = 0.29. Similarly, there were no significant correlations between the three categories of the educational climate and leadership skills or administrative skills of the leader of PGME in the clinical department for neither the trainees' nor the specialists' scores on educational climate. Pearson's correlation coefficients varied between 0.29 (p = 0.07) and 0.03 (p = 0.87).

**Table 2:** Scores on multi-source feedback and educational climate.

	*Mean (SD)*	*Range*
**Multi-source feedback (max score = 7)**		
Total score	5.4 (0.6)	4.2 - 6.3
Leadership performance	5.5 (0.6)	4.2 - 6.3
Management skills	5.2 (0.6)	4.2 - 6.3
		
**Educational climate (max score = 5)**		
Total score*		
All doctors in department	3.0 (0.3)	3.0 - 4,5
Trainees	3.8 (0.3)	3.2 - 4.6
Specialists	4.0 (0.3)	2.6 - 4.5
		
Learning opportunities*		
All doctors in department	3.8 (0.3)	3.3 - 4.4
Trainees	3.7 (0.3)	2,6 - 4,2
Specialists	3.9 (0.3)	2.9 - 4.5
		
Supervision and feedback*		
All doctors in department	3.8 (0.3)	2.6 - 4.7
Trainees	3.7 (0.3)	2.8 - 4,9
Specialists	3.9 (0.3)	2.3 - 4,5
		
Organisation of work		
All doctors in department	4.2 (0.3)	3.2 - 4.7
Trainees	4.1 (0.3)	3.6 - 4.7
Specialists	4,2 (0.4)	2,7 - 4,8

Total mean scores with (SD) and range for a multi-source feedback (MSF) process in clinical consultants responsible for education in clinical departments and a survey on the educational climate among all doctors in the same departments are provided. The MSF scores are divided into two categories: leadership and management skills. The scores on the educational climate are divided into three categories: learning opportunities, supervision and feedback and organization of work. On the educational climate the total scores, trainees' and specialists' scores are provided. *Statistically significant difference between scores from trainees and specialists (p = 0.02).

## Discussion

Surprisingly, this study did not show a significant correlation between the educational climate in clinical departments and the leadership performance of the CRE. There could be various explanations for this finding including both internal and external validity threats.

Firstly, the scores on the educational climate were quite high with a mean score of 3.9 (SD 0.3) on a five-point Likert scale. Moreover, MSF scores on leadership performance were quite high with a mean value of 5.4 (SD 0.6) on a seven-point Likert scale. Both results might indicate an instrumentation bias. However, the scores on the educational climate in our study varied from 3.0 and 4.5. An educational climate score of 3.0 should be considered a low score, since there is a tendency to get positive scores in measurements of educational climate [25,26]. Likewise, respondents in MSF procedures are known to give high scores on MSF [20,22]. The scores on leadership performance ranged between 4.2 and 6.3. A score of 4.2 indicates a rather low leadership performance. The instruments therefore are both able to separate high performers from lower performing CREs and good from a less positive educational climate.

The response rate was moderate 52% (420/811) on the questionnaire on the educational climate and might pose a threat to the validity of these results. In average we got response from ten doctors from each department, which is enough to get a reliable measurement of the educational climate [27].

Finally, we chose to calculate a total MSF score for all respondents. When measuring leadership performance through an MSF procedure you usually separate the respondents into subgroups according to their position in the organisation. Many studies have shown that you perceive the leaders' performance differently according to your position in the organisation (head of department, peer consultant and trainee) [22]. However, in a previous study we have shown that there were only minor differences between the scores of various respondent groups on a MSF process in CREs in clinical departments [20].

The rather high average score in educational climate and MSF scorings might indicate a positive selection bias. However, we excluded the departments where CREs had previously voluntarily signed up for a leadership course and most probably represented the most enthusiastic CREs in the region. Moreover, our study sample included 56 departments covering CREs and departments from a whole region in the country and comprising both university and non-university hospitals in addition to representing many specialties. Even with the lower representation of technical specialties in our study population compared to the background population we feel confident that the results reflect the population in general. Especially since the ratio between the cognitive and surgical specialty departments was the same in the study and the background population. The MSF instrument was developed in a way that only doctors could be invited as respondents. Extending the respondent groups to other staff groups in the department might be considered in future studies in order to achieve a more equal representation of specialties.

In summary, although we acknowledge limitations to our study these do not fully account for our findings. Therefore other explanations to the lack of relation between educational climate and CREs' leadership skills might be speculated, including organisational issues of PGME.

In one way PGME relates to a parallel organisation outside the organisation of hospitals and other health care organisations where PGME takes place [6,28]. PGME is governed by outside bodies like a national boards of health (United Kingdom, Denmark)[15,29], the Accreditations Councils (USA) [17], specialist societies (Canada) [18] or the union (Norway) [30]. Contrary to this the CRE refers to the head of department in administrative matters. This unclear line of reference combined with an unclear task description may contribute to the CRE's low impact [19]. The hospitals are run administratively through the heads of departments. This places the CRE in a position as middle manager primarily concerned with interpreting and implementing policies and programs from the educational bodies or places him as a low-level manager supposedly engaged in structuring, coordinating and facilitating work activities [24]. In a previous study we have shown that the CRE is expected to manage a whole range of administrative duties [19], among them ensuring that trainees are exposed to the clinical situations they are supposed to learn from. Structuring the learning opportunities and processes is very important in clerkship education [31,32], and there is reason to believe that it might be even more important in PGME, where involvement, participation and interpersonal relations are so fundamental [33]. Since stakeholders in PGME found that the CRE masters the administrative duties well [19], we would have expected a relation between the organisation of work and management skills of the CRE. Finding no relation might reflect that the CRE have limited influence on the planning of daily work schedules. Both CREs and stakeholders have suggested the development of a specific leadership course for CREs to strengthen their position [19]. However, if the CRE is in a weak position to influence the working organisation and educational climate these initiatives may be in vain [34].

CREs might be fairly good leaders but acting in a system that makes it difficult to be perceived as a leader of education and creator of the educational culture. Additionally, the educational climate might be so mixed up in the work environment that maybe focus should be on the working culture instead of isolating the educational climate. This would involve asking other staff groups about their perception of the working culture in the clinical departments.

To further explore factors that influence the educational climate it might be relevant to focus on the leadership performance of the administrative heads of the clinical departments. In particular how the head of the department prioritises PGME and attends to the educational mission in the department. This might have significant influence on the CRE's possibility to excert leadership of education and fulfil expectations [24].

## Conclusion

Our results indicate that there is no relationship between the educational climate in clinical departments and the leadership performance of educational leaders of PGME in the department. The separated administrative and educational lines of reference in PGME might explain this lack of relation. Future studies should focus on exploring how administrative leaders of clinical departments and perhaps other factors related to the clinical organisation influence the educational climate.

## List of abbreviations

PGME: Postgraduate medical education; MSF: Multi-source feedback; CRE: Consultant responsible for education in clinical department; DNBH: Danish National board of Health; SD: Standard deviation; PHEEM: Postgraduate hospital educational environment measure

## Competing interests

The authors declare that they have no competing interests.

## Authors' contributions

BM and LM made substantial contributions to the conception, design and the acquisition of data. BM analyzed the data. BM, LSM, AJJS and CR made substantial contribution to the interpretation of data and drafting of the manuscript. BM, LSM, AJJS and CR all made substantial contributions in critically revising the manuscript and content. All authors have given final approval of the version published.

## Authors' information

BM: MD, MHPE and associate professor in postgraduate medical education is director for specialist training at Aarhus University Hospital, Skejby, Denmark.

LSM: MD, PhD and associate professor in postgraduate medical education is consultant at the Department of Internal Medicine and director for specialist training at the Regional Hospital, Viborg, Denmark.

AJJS: MD, PhD is professor of medical education and scientific director of the Institute for Education, Faculty Health, Medicine and Life Sciences, Maastricht University, Netherlands.

CR: MD, PhD, MHPE is professor of medical education and director of Centre for Clinical Education, Copenhagen University and Capital Region, Rigshospitalet, Denmark.

## Pre-publication history

The pre-publication history for this paper can be accessed here:

http://www.biomedcentral.com/1472-6920/10/62/prepub
